# A gossypiboma (foreign body granuloma) mimicking a residual odontogenic cyst in the mandible: a case report

**DOI:** 10.1186/1752-1947-5-211

**Published:** 2011-05-28

**Authors:** Guido R Sigron, Michael C Locher

**Affiliations:** 1 Department of Oral Surgery, Center for Dental and Oral Medicine and Cranio-Maxillofacial Surgery, University of Zurich, Plattenstrasse 15, 8032 Zurich, Switzerland

## Abstract

**Introduction:**

Gossypiboma (foreign body granuloma) in the tooth socket as a complication of tooth removal is rare. Several cases of gossypiboma have been reported after orthopedic, abdominal, otorhinolaryngology, or plastic surgery, but there has been only one reported case after oral surgery.

**Case presentation:**

A 42-year-old Caucasian German-speaking Swiss woman applied to our clinic for removal of her right mandibular first molar. Her right mandibular third molar had been removed seven years ago. Post-operatively, she complained of pain and foreign body sensation for six months in the area of the removed tooth. A panoramic radiograph of our patient showed a defined and oval radiolucent area in the socket of the right mandibular third molar evoking a residual cyst. An operation was planned to remove the cyst-like lesion. During surgery, a foreign body composed of gauze was found in the right mandibular third molar region. The histological findings were compatible with a foreign body reaction around gauze.

**Conclusion:**

Retained gauze must be considered if patients complain of pain and foreign body sensation after tooth removal. The use of gauze with radio-opaque markers and extensive irrigation of the socket with saline to remove gauze fragments can avoid this mishap.

## Introduction

The removal of lower third molars is one of the most frequently performed oral surgical procedures [1]. After the third molar has been extracted and the socket has been treated, the envelope (sulcular) mucoperiosteal flap or triangular flap is repositioned. Three wound-healing techniques following lower third molar removal exist: primary closure alone, primary closure with drainage and open healing with a dressing. For drainage or dressing, gauze, such as an iodoform-vaseline drain (IVD), can be used. At the follow-up appointment, the sutures and the wound dressing are removed. If the removal of the wound dressing is forgotten, a wound-healing disorder or foreign body reaction can occur. A retained surgical gauze (sponge) is called a gossypiboma or textiloma. The term 'gossypiboma' is derived from the combination of the Latin word 'gossypium' for 'cotton' and the Swahili word 'boma' for 'place of concealment' [2]. Several cases of gossypiboma have been reported after orthopedic [3,4], abdominal [5-7], otorhinolaryngology [8,9], or plastic surgery [2], but there has been only one reported case after oral surgery [10]. To the best of our knowledge, this is the first case in which a foreign body was forgotten in the socket of a third molar for seven years. We will discuss diagnosis, clinical management, and medical-legal implications.

## Case presentation

**Figure 1:**
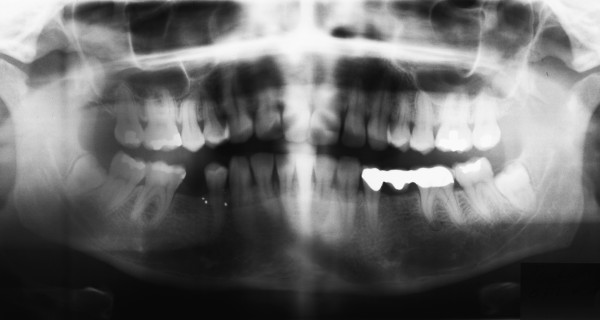
**Pre-operative panoramic radiograph of the right mandibular third molar in place with no cyst-like radiolucency**.

A 42-year-old Caucasian German-speaking Swiss woman applied to our clinic for removal of her right mandibular first molar. Our patient reported receiving a dental implant in her right premolar region seven years ago. During this surgery, the dentist also removed the right mandibular third molar (Figure 1). Post-operatively, she complained of pain and foreign body sensation for six months in the area of the removed tooth. The dentist undertook several measures but decided against an active treatment and, therefore, the medical problems did not improve. Our patient herself started oral irrigation with marigold tea, leading to relief of pain and foreign body sensation.

**Figure 2:**
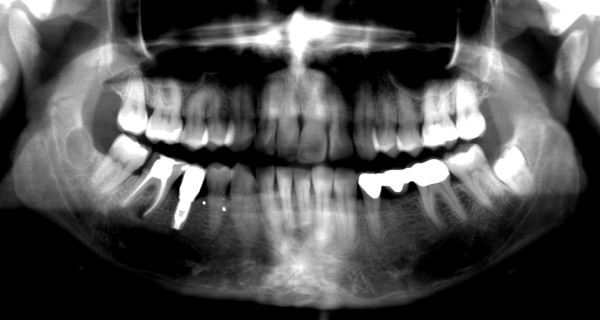
**Panoramic radiograph showing the cyst-like radiolucency in the right mandibular third molar region**. Seven years after removal of the right mandibular third molar.

**Figure 3:**
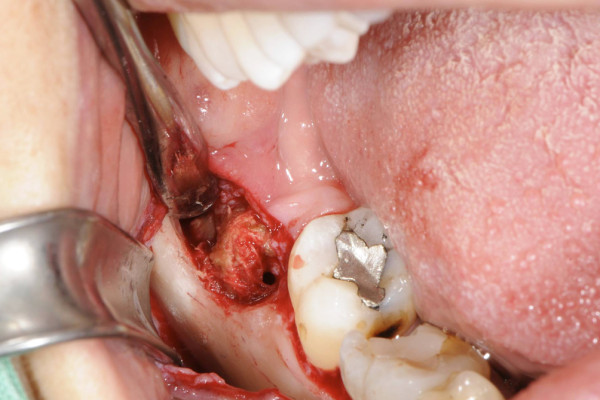
**Operative findings show the retained IVD in the right mandibular third molar region**.

**Figure 4:**
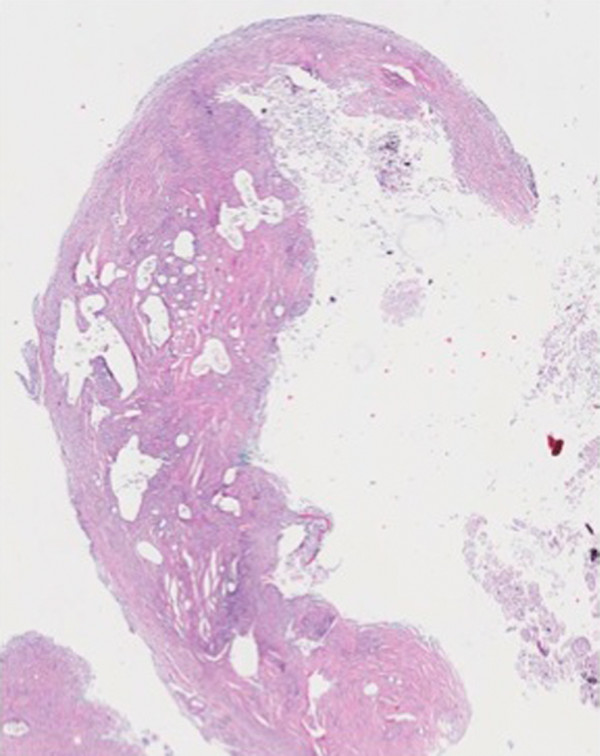
**Soft tissue specimen with numerous empty spaces of varying size containing fragments of foreign material**. Photomicrograph ×25. Hematoxylin and eosin.

**Figure 5:**
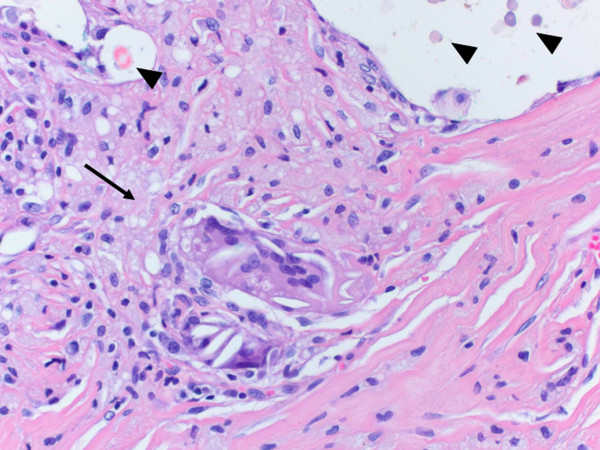
**Granulomatous inflammation (arrow) and foreign bodies within empty spaces (arrowheads)**. Photomicrograph ×400. Hematoxylin and eosin.

**Figure 6:**
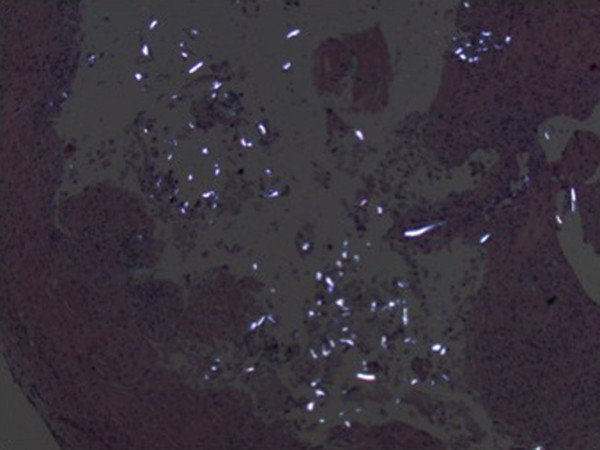
**Numerous birefringent foreign bodies under polarized light**. Photomicrograph ×200.

At her first visit to our clinic, an oral examination showed an insufficient composite filling on her lower right first molar, which was fractured on the distal side. There was no tenderness or percussion sensitivity in her teeth, nor was there swelling or erythema of the gingiva and oral mucosa. There were also normal periodontal circumstances. A panoramic radiograph showed a defined and oval radiolucent area in the socket of her right mandibular third molar (Figure 2). The features in this radiograph could suggest a diagnosis of residual cyst, keratocyst, odontogenic cyst, or unicystic ameloblastoma. There was no relationship between the oval radiolucent area and the inferior alveolar canal, so a computed tomography (CT) scan or cone-beam CT (CBCT) was not necessary. The treatment consisted of a total surgical excision, in which the cyst-like lesion was removed. In addition, her right mandibular first molar was extracted. During surgery, a foreign body composed of gauze was found in the oval radiolucent area (Figure 3). There was no infection or abscess formation around the gauze. The mass was completely removed from the bone and sent for histological diagnostic examination. The histological examination of the tissues around the gauze revealed aseptic chronic inflammatory infiltration and granuloma formation with birefringent foreign bodies, compatible with gauze fragments (Figures 4, 5, 6). No cyst epithelium was found by microscopy or immunohistochemical tests with AE1/3. The histopathologic diagnosis was foreign body reaction around birefringent foreign bodies and calcifications.

Our patient's post-operative course was uneventful; complete recovery of the retromolar area was noted on the follow-up examination after two weeks.

## Discussion

CT and CBCT scans are the most effective methods to diagnose gossypiboma, showing a round, low-intensity, ill-defined mass containing a spongiform air bubble. Ultrasonography is another diagnostic method, showing echogenic masses with intense and sharply delineated acoustic shadows or hypoechogenic masses with complex echogenic foci [6]. If the gauze contains a radiographically detectable material, such as an iodoform or a radio-opaque filament, a gossypiboma is easy to diagnose [11]. When no radio-opaque marker is seen on X-ray, CBCT, or CT scans, the characteristic internal structure of the gauze granuloma is best visualized using magnetic resonance imaging (MRI) [5]. Bone scintigraphy does not necessarily provide additional useful information for differentiation [4]. A definitive diagnosis requires a histological examination of the removed pathological tissue. Two types of reactions to foreign bodies are described in pathology: the exudative type, leading to abscess formation or, very rarely, an aseptic fibrinous response, which results in adhesion or encapsulation, leading to granuloma formation [12].

When the pathological tissue shows only a chronic inflammatory lesion with foreign body giant cells, without many birefringent foreign bodies, the diagnosis is oral pulse granuloma. Oral pulse granuloma is most commonly found in the posterior regions of the mandible, so there is an important differential diagnosis in that case [13]. In the literature, foreign body reactions are also described after injection of biomaterials or around hemostatic materials, mimicking recurrent tumors on MRI [3,14].

Gauze or IVDs are not safe because they can break into fragments during manipulations [3]. Therefore, it is especially important to flush the socket extensively with saline and to check for foreign materials. When the patient reports subjective symptoms such as foreign body sensation, the operative site should be controlled. If retained gauze is detected in the socket, the patient should be informed and asked for permission for a second surgical procedure.

Of course, a post-operative infection should be covered by the pre-operative informed consent of the patient. Otherwise, the patient can bring a civil lawsuit against the surgeon for surgical complications. Critical points are negligent bodily harm and surgery-related co-morbidities, such as psychological pain from prolonged treatment and infectious complications [7].

## Conclusion

Retained gauze must be considered if patients complain of pain and foreign body sensation after tooth removal. This case emphasizes the importance of the follow-up appointment with removal of sutures and gauze. The use of gauze with radio-opaque markers and extensive irrigation of the socket with saline to remove gauze fragments can avoid this mishap. Despite proper management, human errors cannot be completely eliminated.

## Abbreviations

CBCT: cone-beam CT; CT: computed tomography; IVD: iodoform-vaseline drain; MRI: magnetic resonance imaging

## Consent

Written informed consent was obtained from the patient for publication of this case report and any accompanying images. A copy of the written consent is available for review by the Editor-in-Chief of this journal.

## Competing interests

The authors declare that they have no competing interests.

## Authors' contributions

GRS was a major contributor in writing the manuscript, and gathering and analyzing the data regarding the history and the operation of our patient. MCL provided clinical insights and final approval for the manuscript as the head of the department. All authors read and approved the final manuscript.

## References

[B1] Sailer HF Pajarola GF Oral surgery for the general dentist 1998 New York: Thieme

[B2] Song SY Hong JW Yoo WM Tark KC Gossypiboma after mandibular contouring surgery J Craniofac Surg 2009 20 1607 1610 10.1097/SCS.0b013e3181b14761 19816308

[B3] Okten AI Adam M Gezercan Y Textiloma: a case of foreign body mimicking a spinal mass Eur Spine J 2006 15 Suppl 5 626 629 16736201 10.1007/s00586-006-0136-6 PMC1602194

[B4] Sakayama K Fujibuchi T Sugawara Y Kidani T Miyawaki J Yamamoto H A 40-year-old gossypiboma (foreign body granuloma) mimicking a malignant femoral surface tumor Skeletal Radiol 2005 34 221 224 10.1007/s00256-004-0821-7 15365779

[B5] Bani-Hani KE Gharaibeh KA Yaghan RJ Retained surgical sponges (gossypiboma) Asian J Surg 2005 28 109 115 10.1016/S1015-9584(09)60273-6 15851364

[B6] Cevik I Dillioglugil O Ozveri H Akdas A Asymptomatic retained surgical gauze towel diagnosed 32 years after nephrectomy Int Urol Nephrol 2008 40 885 888 10.1007/s11255-008-9383-2 18443914

[B7] Schmid C Krempel S Scheld HH A forgotten gauze swab--clinical and legal considerations Thorac Cardiovasc Surg 2001 49 191 193 10.1055/s-2001-14298 11432483

[B8] Amr AE A submandibular gossypiboma mimicking a salivary fistula: a case report Cases J 2009 2 6413 10.4076/1757-1626-2-6413 19829801 PMC2740320

[B9] Ozer C Ozer F Sener M Yavuz H A forgotten gauze pack in the nasopharynx: an unfortunate complication of adenotonsillectomy Am J Otolaryngol 2007 28 191 193 10.1016/j.amjoto.2006.07.009 17499138

[B10] Pons Y Schouman T Maxillary sinus textiloma: a case report J Med Case Reports 2010 4 288 20735833 10.1186/1752-1947-4-288 PMC2936320

[B11] Catterlin RK Throndson RR Iodoform gauze with a radiopaque filament Oral Surg Oral Med Oral Pathol 1993 76 257 8361742 10.1016/0030-4220(93)90215-p

[B12] Prasad S Krishnan A Limdi J Patankar T Imaging features of gossypiboma: report of two cases J Postgrad Med 1999 45 18 19 10734327

[B13] Philipsen HP Reichart PA Pulse or hyaline ring granuloma. Review of the literature on etiopathogenesis of oral and extraoral lesions Clin Oral Investig 2010 14 121 128 10.1007/s00784-009-0322-0 19714375

[B14] Jham BC Nikitakis NG Scheper MA Papadimitriou JC Levy BA Rivera H Granulomatous foreign-body reaction involving oral and perioral tissues after injection of biomaterials: a series of 7 cases and review of the literature J Oral Maxillofac Surg 2009 67 280 285 10.1016/j.joms.2008.01.052 19138600

